# Viral Co-infection and Leprosy Outcomes: A Cohort Study

**DOI:** 10.1371/journal.pntd.0003865

**Published:** 2015-08-12

**Authors:** Paulo R. L. Machado, Lídia M. Machado, Mayume Shibuya, Jamile Rego, Warren D. Johnson, Marshall J. Glesby

**Affiliations:** 1 Immunology Service, Federal University of Bahia, Salvador, Bahia, Brazil; 2 Instituto Nacional de Ciência e Tecnologia de Doenças Tropicais, Salvador, Bahia, Brazil; 3 Department of Medicine, Center for Global Health, Weill Cornell Medical College, New York, New York, United States of America; 4 Department of Medicine, Division of Infectious Diseases, Weill Cornell Medical College, New York, New York, United States of America; Center for Disease Control and Prevention, UNITED STATES

## Abstract

**Background:**

The role of the host immunity in determining leprosy clinical forms and complications is well recognized, implying that changes in the immune status may interfere with several aspects of the disease. Therefore, we hypothesized that the presence of viral co-infections and associated immunological changes will have a clinical impact on leprosy outcomes. The aim of our study was to determine the clinical impact of human immunodeficiency virus (HIV), human T cell lymphotrophic virus type 1 (HTLV-1), hepatitis B virus (HBV) and hepatitis C virus (HCV) co-infection on the development of reactions, neuritis, neuropathy and relapses.

**Methodology/Principal Findings:**

Cohort study in 245 leprosy subjects from Bahia, Brazil. Patients were followed from the time of diagnosis until at least the end of multidrug therapy. Viral co-infection was detected in 36 out of the 245 patients (14.7%). Specific co-infection rates were 10.6% for HBV, 2.9% for HIV, 2.5% for HTLV-1 and 0.8% for HCV. All four groups of co-infected patients had higher rates of neuritis and nerve function impairment compared to non co-infected leprosy subjects. The relapse rate was also higher in the co-infected group (8.3%) versus patients without co-infection (1.9%); relative risk 4.37, 95% confidence interval 1.02–18.74.

**Conclusions/Significance:**

Leprosy patients should be screened for HBV, HCV, HIV and HTLV-1 co-infections. Besides contributing to better health care, this measure will facilitate the early detection of severe complications through targeting of higher risk patients.

## Author Summary

The clinical and social impact of leprosy, a disease caused by *Mycobacterium leprae*, is due to the deformities and disabilities that are consequences of skin and peripheral nerve inflammation. We believe that leprosy patients who also have an associated viral infection will have a worse outcome. This can be due to alterations in the immune system triggered by the virus co-infection, leading to an increasing inflammation of the skin or peripheral nerves affected by *Mycobacterium leprae*, or to a depression of the mechanisms that control the infection. We have verified if the presence of specific viral infections in leprosy subjects is associated with a higher risk of developing a worse outcome like nerve inflammation, impairment in the nerve function or relapse. We have screened the patients for human immunodeficiency virus, human T cell lymphotrophic virus type 1, hepatitis B and hepatitis C virus. These subjects were followed up during all the period of leprosy treatment (at least 6 months) in order to detect the emergence of any complication. We have found that leprosy patients with viral co-infection are at higher risk to develop nerve inflammation and damage, as well as relapse, compared to subjects without viral co-infection.

## Introduction

The host immune response against *Mycobacterium leprae* is closely related to leprosy spectrum and clinical outcome [1]. During the chronic course of the disease about 40% of the patients may develop acute inflammatory episodes–leprosy reactions–often associated neuritis that may lead to neuropathy and deformities [2, 3]. These complications have an important impact on the patient’s health along with a major psychological, social and economic burden. Additionally, these reactions necessitate long-term treatment with drugs such as corticosteroids, thalidomide, and immunosuppressive agents [4] that are associated with many side effects and increasing morbidity.

The two types of leprosy reactions, type 1 reaction (T1R) and type 2 reaction (T2R) are immune mediated. T1R is associated with an exacerbated cellular response with increased *in situ* Th1 cytokine production, whereas T2R is associated with elevated peripheral production of inflammatory chemokines and cytokines like IL-6 and TNFα, immune complex deposits and neutrophil infiltration in tissues [1, 5, 6].

Co-infections in leprosy may modify the host immunity either by enhancing inflammation and tissue damage leading to reactions and neuritis [7], or depressing defense mechanisms resulting in higher bacterial load or relapses [8].

The aim of this study was to determine if specific viral co-infections by human immunodeficiency virus (HIV), human T cell lymphotrophic virus type 1 (HTLV-1), hepatitis B virus (HBV) and hepatitis C virus (HCV) are associated with leprosy unfavorable outcomes. The primary clinical outcomes were the emergence of reactions, neuritis, neuropathy and relapses.

## Materials and Methods

### Study design

This cohort study was performed in the outpatient clinics from two leprosy referral centers in Salvador, Brazil, the Hospital Universitário Prof. Edgar Santos of the Federal University of Bahia and the Hospital Dom Rodrigo de Menezes. A total of 245 patients were included and followed until at least the end of multidrug therapy (MDT) or 6 months post-enrollment. The patients were enrolled from October 2010 to June 2013.

### Inclusion criteria

Eligible subjects were either newly diagnosed with leprosy, already under MDT, or in follow up after completion of MDT. All patients were classified by the Ridley-Joplin score and by the WHO field classification [9, 10].

### Study procedures

#### Outcome definitions

T1R: acute onset of erythema and edema of cutaneous lesions associated or not with neuritis and edema of hands, feet or face. T2R or erythema nodosum leprosum (ENL): acute onset of subcutaneous nodules anywhere in the body associated or not with neuritis, fever, malaise, myalgia, or other systemic symptoms. Neuritis: acute nerve thickening and pain. Nerve function impairment (NFI): a reduction in sensory or motor function associated with WHO disability grades type 1 or 2 [10]. Silent neuropathy: presence of NFI without symptoms like peripheral nerve pain or thickening. Unfavorable outcome: presence of reactions, neuritis or silent neuropathy. Relapse: WHO criteria were used for MB and PB disease [11]. For MB leprosy, a marked increase (at least 2+ over the previous value) in the bacillary index (BI) at any single site, usually with evidence of clinical deterioration (new skin patches or nodules and ⁄ or new nerve damage). For PB disease, new skin lesions without definite improvement within four weeks of corticosteroid therapy.

#### Serology

Blood was drawn to test for viral co-infections at the time of enrollment in the study. HIV–Analysis for HIV-1 and HIV-2 antibodies was performed by enzyme linked immuno sorbent assay (ELISA) with Murex HIV Ag/Ab combination (DiaSorin, Dartfort, UK). Confirmatory testing was done with Western Blot analysis. HTLV–Sera with HTLV-1 diagnosis by ELISA (Cambridge Biotech Corp., Worcester, MA) were confirmed by Western Blot analysis. HBV–Hepatitis B core antigen (HBc) total antibodies was determined using an Anti-HBc Architect System (Abbott Laboratories. Abbott Park, Illinois, U.S.A). HCV–HCV IgG antibodies was determined using an Anti-HCV Architect System (Abbott Laboratories. Abbott Park, Illinois, U.S.A). HCV RNA was determined by RealTime PCR (Abbot Laboratories. Abbott Park, Illinois, U.S.A).

#### Statistical analysis

Statistical tests were performed with GraphPad Prism 6.00 (Graphpad Software Inc. La Jolla, CA, USA). For the analysis of quantitative data, means, medians, and standard deviations (SDs) were calculated. Fisher’s exact test was used to compare categorical data, and relative risks (RR) with 95% confidence intervals (CI) were calculated. The non-co-infected group of subjects served as the reference group for all comparisons. In the analysis of quantitative data, unpaired t-test was used whenever the distribution of variables was normal. Log-rank (Mantel-Cox) test was used to compare survival curves. The significance level was defined as P < 0.05. P-values were not adjusted for multiple comparisons.

#### Sample size

An estimated sample size of 280 patients was calculated with 80% power to detect a 10% difference rate between any co-viral infection in patients that develop unfavorable outcome compared with patients that do not develop unfavorable outcome.

#### Ethical aspects

Written informed consent was obtained from all subjects enrolled. The study was approved by the Institutional Review Boards of the Hospital Universitário Prof. Edgar Santos from the Federal University of Bahia, Hospital Dom Rodrigo de Menezes, and the Weill Cornell Medical College.

## Results

A total of 273 leprosy patients were enrolled in the study; 28 were excluded due to loss to follow-up (20 subjects), or after moving to another city (8 subjects). From the remaining 245 subjects, 192 (78%) were newly diagnosed cases and 53 patients (22%) were already under treatment and follow-up; twelve subjects were receiving MDT, including three patients with relapse, while the others (41 subjects) had already finished MDT but under treatment for long standing reactions or neuritis. All subjects were prospectively followed for at least 6 months. Demographic, clinical and laboratorial data from the 245 participants are presented in Table 1.

**Table 1:** Demographic, clinical and laboratory characteristics in a cohort of leprosy patients according to the presence or absence of viral co-infection.

	Without co-infection	With co-infection^1^	HBV co-infection	HIV co-infection	HTLV-1 co-infection	HCV co-infection	P-value
	N = 209 (%)	N = 36 (%)	N = 26 (%)	N = 7 (%)	N = 6 (%)	N = 2 (%)	
*Gender*							
Male	127 (60.8)	21 (58.3)	18 (69.2)	3 (42.8)	1 (16.6)	2	0.04* ^#^
Female	82 (39.2)	15 (41.7)	8 (30.8)	4 (57.2)	5 (83.4)	0	
*Age*							
Range	18–68	26–65	27–65	26–44	37–65	52–57	0.0043** ^Ŧ^
Mean ± SD	41.7±13	48.4±11.9	50.1±12	36.4±5.7	47.5±10.6		0.002** ^##^
*Clinical form* ^*2*^							
I	20 (9.6)	3 (8.3)	1 (3.8)	1 (14.5)	2 (33.3)	0	
N	5 (2.4)	1 (2.8)	0	0	1 (16.7)	0	
TT	29 (13.9)	5 (13.9)	5 (19.2)	0	0	0	
BT	29 (13.9)	8 (22.2)	6 (23.1)	3 (42.8)	1 (16.7)	1 (50)	
BB	28 (13.4)	7 (19.4)	5 (19.2)	1 (14.5)	1 (16.7)	0	
BL	36 (17.2)	4 (11.1)	4 (15.4)	0	0	0	
LL	62 (29.7)	8 (22.2)	5 (19.2)	2 (28.6)	1 (16.7)	1 (50)	
*WHO classification*							
Paucibacillary	76 (36.4)	13 (36.1)	10 (38.5)	3 (42.8)	2 (33.3)	1 (50)	ns*
Multibacillary	133 (63.6)	23 (63.9)	16 (61.5)	4 (57.2)	4 (66.7)	1 (50)	
*Follow up* (months)							
Range	18–68	6–107	6–107	10–60	12–60	16–107	ns**
Mean ± SD	28.6±22.2	30±20.7	30.6±22.3	27.4±20.4	36.5±18.4	61.5±64.3	0.04** ^###^
*BI* ^*3*^							
Range	0–6	0–5	0–4.75	0–5	0–4	0–4.5	ns**
Mean ± SD	1.6±1.8	1.4±1.9	1.4±1.9	1.4±2.2	0.9±1.6	1.4±1.9	

^1^ Three subjects had HIV & HTLV-1 coinfection, 1 had HIV & HBV, and 1 had HBV and HCV

^2^ Indeterminate (I), pure neural (N), tuberculoid (TT), borderline-tuberculoid (BT), borderline-borderline (BB), borderline-lepromatous (BL), lepromatous (LL)

^3^ BI, bacillary index

* Fisher’s exact test

** Unpaired t test

Ŧ Comparison between the group without co-infection and the group with co-infection

# Comparison between the group HTLV-1 co-infection and the group without co-infection

## Comparison between the group HBV co-infection and the group without co-infection

### Comparison between the group HCV co-infection and the group without co-infection

The overall rate of viral co-infection was 14.7%. The most common co-infection was HBV (26/245, 10.6%), while HIV, HTLV-1 and HCV co-infection rates were 2.9%, 2.5% and 0.8% respectively. Three subjects were infected with both HIV and HTLV-1, one with both HIV and HBV and another one with both HCV and HBV.

The majority of subjects were male (60.8%). The male:female ratio was reversed in the HIV and HTLV-1 groups with a higher number of cases in females. Ages ranged from 18 to 68 years old, with the co-infected group and the HBV group tending to have older subjects. The majority of subjects (64%) were classified as MB and the remainders were PB. The higher proportion of MB cases was also observed among the co-infected groups. Borderline forms and LL pole were diagnosed in 45.7% and 28.7% of the 245 cases respectively. The follow up period ranged from 6 to 107 months (28.9 ± 22.3) and was similar in the groups with or without viral co-infection but higher in the two subjects co-infected with HCV (Table 1).

Table 2 shows the clinical outcome in subjects with or without any viral co-infection. In the group of patients with co-infection, T1R, T2R or neuritis were detected in 80.5%, compared to 68.9% of subjects without co-infection (RR 1.30, CI 1.12–1.49). The occurrence of both T1R and T2R in the same patient at different times was diagnosed in 8.3% of co-infected subjects and 2.4% of patients without co-infection.

**Table 2:** Clinical outcomes in a cohort of leprosy patients according to the presence or absence of viral co-infection.

	Without co-infection	With co-infection	HBV co-infection	HIV co-infection	HTLV-1 co-infection	HCV co-infection	P-value
	N = 209 (%)	N = 36 (%)	N = 26 (%)	N = 7 (%)	N = 6 (%)	N = 2 (%)	
Reactions							
*Type 1*	52 (24.9)	10 (27.8)	8 (30.8)	3 (42.8)	1 (16.7)	1 (50)	
*Type 2*	68 (32.5)	7 (19.4)	4 (15.4)	2 (28.6)	1 (16.7)	1 (50)	
*Type 1 and 2*	5 (2.4)	3 (8.3)	2 (7.7)	0	1 (16.7)	0	
*Any*	125 (59.8)	20 (55.5)	14 (53.9)	5 (71.4)	3 (50)	2 (100)	ns*
Neuritis	19 (9.1)	9 (25)	7 (26.9)	1 (14.3)	2 (33.3)	0	0.01* ^Ŧ^; 0.01* ^##^
Reactions and neuritis	144 (68.9)	29 (80.5)	21 (80.8)	6 (85.7)	5 (83.3)	2 (100)	0.01* Ŧ
NFI^1^							
*Associated* ^*2*^	70/186 (37.6)	16/33 (48.5)	11/23 (47.8)	4 (57.1)	4 (66.6)	1 (50)	ns*
*Silent neuropathy*	3/186 (1.6)	4/33 (12.1)	3/23 (13)	0	1 (16.6)	0	0.01* ^Ŧ^; 0.02* ^##^
*Total*	73/186 (39.2)	20/33 (60.6)	14/23 (60.8)	4 (57.1)	5 (83.3)	1 (50)	0.03* ^Ŧ^; 0.04* ^#^
Unfavorable outcome^3^	147 (70.3)	33 (91.7)	24 (92.3)	6 (85.7)	6 (100)	2 (100)	0.007* Ŧ; 0.0018* ^##^
Relapses	4 (1.9)	3 (8.33)	2 (7.69)	1 (14.28)	1 (16.6)	0	ns*

^1^ NFI: nerve function impairment

^2^ Associated with reaction or neuritis

^3^ Reactions, neuritis or silent neuropathy

* Fisher’s exact test

Ŧ Comparison between the group without co-infection and the group with co-infection

# Comparison between the group HTLV-1 co-infection and the group without co-infection

## Comparison between the group HBV co-infection and the group without co-infection

**Fig 1:**
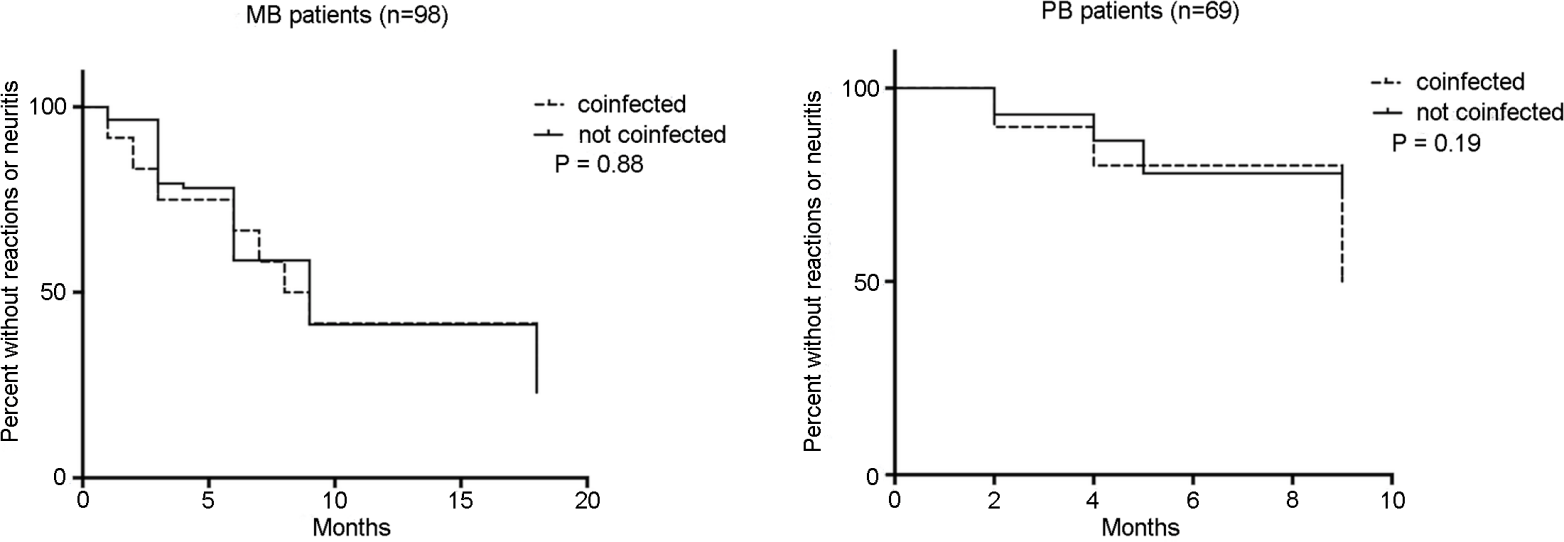
Probability of being free of developing reactions or neuritis during and after multidrug therapy.

Overall, the incidence of reactions and neuritis was 39.2% in patients before and 46.7% during MDT, and this did not differ based on co-infection status. The incidence of these complications over time during and after MDT also did not differ by presence of co-infection when stratified by PB and MB disease types (Fig 1).

Silent neuropathy was found in 12.1% and 1.6% of co-infected and non-co-infected subjects respectively (RR 7.52, CI 1.76–32.05). Nerve function impairment (NFI) was documented in 60.6%of subjects with co-infection compared to 39.2% without co-infection (RR 1.54, CI 1.11–2.14).

The rates of unfavorable outcomes among those with and without viral co-infection were 91.7% and 70.3%, respectively (RR 1.30, CI 1.14–1.49). The relapse rate was 8.3% in patients with co-infection compared to 1.9% in those without co-infection (RR 4.37, CI 1.02–18.74).

### HBV co-infection

In this group the presence of T1R, T2R and neuritis was detected in 80.7%, silent neuropathy in 13%, and NFI in 60.8%, for a total of 93.7% with unfavorable outcomes (RR 1.31, CI 1.14–1.51). The relapse rate was 7.7% (RR 4.04, CI 0.77–20.98).

### HIV co-infection

In the seven subjects with HIV co-infection, either T1R, T2R or neuritis was diagnosed in 85.7%, and no silent neuropathy was detected. NFI was found in 57%, and an unfavorable outcome in 83.3%. Five patients had previously known HIV diagnoses and were on combination antiretroviral therapy; four of these 5 developed reactions or neuritis. Additionally, 3 out of the HIV-infected subjects were also co-infected with HTLV-1. Of these three subjects, one PB case developed a T1R and relapsed, while the others each developed a T2R or neuritis.

### HTLV co-infection

**Fig 2:**
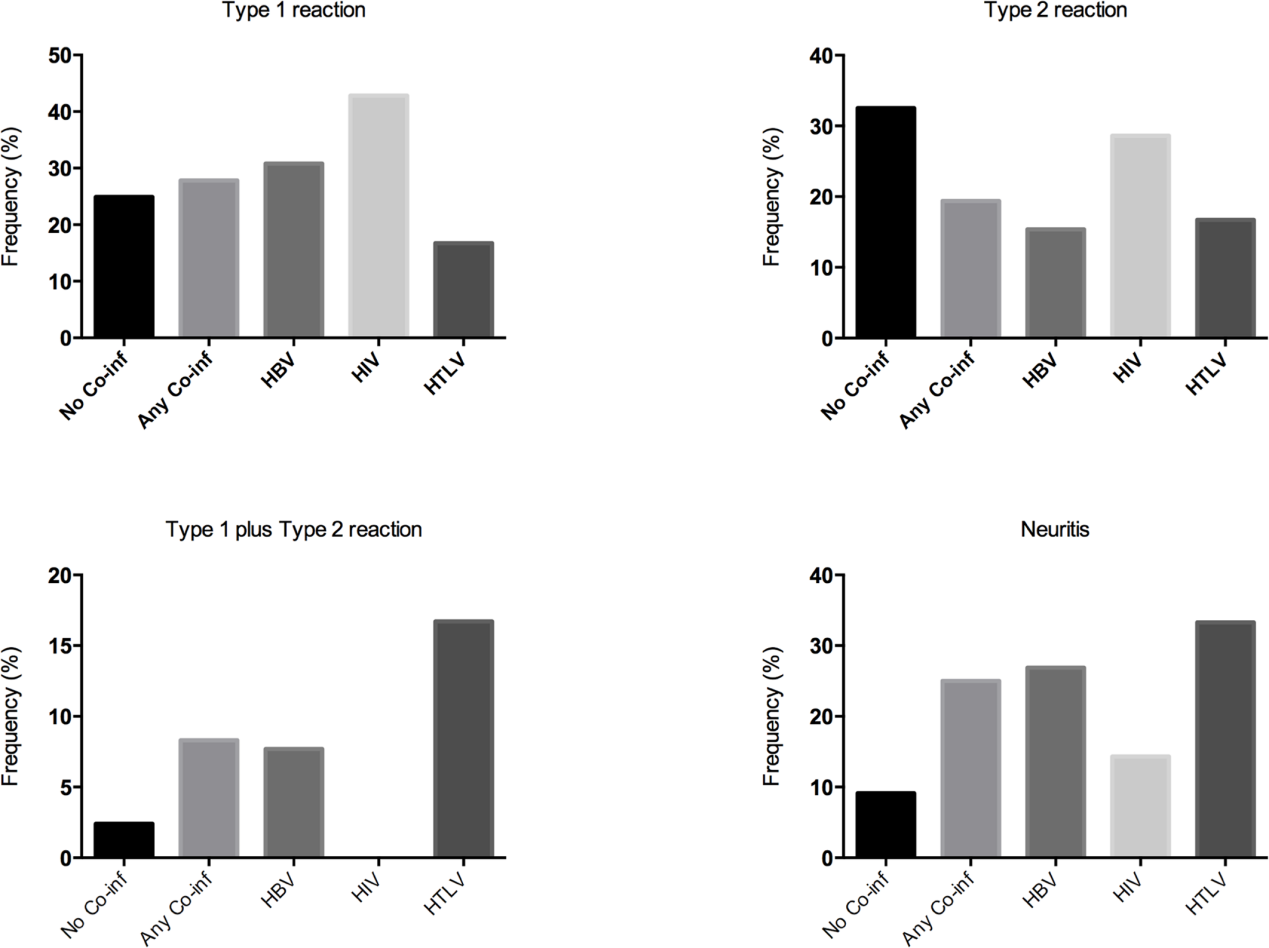
Frequencies of type 1 reaction, type 2 reaction, type 1 plus type 2 and neuritis among co-infected and not co-infected leprosy patients.

Among those with HTLV-1 infection, T1R, T2R and neuritis were diagnosed in 83.3%, silent neuropathy in 16.6%, and all six subjects had unfavorable outcomes (RR 1.42, CI 1.30–1.55). Five out of six patients developed NFI (RR 2.12, CI 1.42–3.17). Half of the HTLV-1 cases also had HIV co-infection as described above. Higher frequencies of T1R plus T2R, as well as neuritis were found in HTLV-1 co-infected patients (Fig 2).

None of the HTLV-1 co-infected subjects had specific diseases associated with this virus, such as adult T-cell lymphoma/leukemia, or HTLV-1-associated myelopathy / tropical spastic paraparesis.

### HCV co-infection

Two of 245 subjects were HCV seropositive. One patient who was also co-infected with HBV developed a T1R associated with NFI, and the other a T2R. No relapses were documented.

## Discussion

Reactions and neuritis are the main cause of peripheral nerve damage in leprosy. An important strategy to avoid tissue and nerve damage is early diagnosis and prompts management of complications. The identification of risk factors for adverse leprosy outcomes may facilitate earlier diagnosis and lead to improved clinical care. Although the main underlying mechanisms and causes of these complications are not well understood, some studies suggest that MB forms of disease, high BI, initiation of MDT, vaccination, and emotional stress, among others, may be considered as risk factors [12, 13, 14]. It is hypothesized that these factors interfere with the host immune response leading to tissue inflammation. Some observations suggest that infectious diseases may influence leprosy clinical manifestations. For instance, the presence of intestinal parasites, which drives a Th2 pathway, was associated with lepromatous disease [8] and chronic oral infections with reactions [15].

The role of viral co-infections in leprosy remains unknown. One of the most studied viruses in terms of seroprevalence in leprosy is HBV, after an initial report by Blumberg et al. study describing an association between HBV infection and lepromatous leprosy [16]. Since then, numerous studies [17] have confirmed or refuted this association. Although a higher HBV prevalence among institutionalized patients with leprosy has been found in Brazil, HBV co-infection was not clearly associated with any clinical leprosy outcome [18, 19, 20]. A similar situation has been found regarding HCV co-infection in leprosy, where a higher HCV prevalence was associated with institutionalization and occasionally with lepromatous disease [21, 22]. Our study was conducted in outpatients and although we did not find any specific leprosy clinical form associated with HBV or HCV co-infections, we showed that HBV co-infection was associated with a higher rate of neuritis and NFI, whereas the two subjects with HCV co-infection presented reactions. Interestingly, a previous study from our group suggested that these viruses were related to a higher T1R prevalence [23].

The interaction between leprosy outcome and HIV infection is more evident during the development of the immune reconstitution inflammatory syndrome (IRIS) in patients on combination antiretroviral therapy. In this situation, subclinical leprosy is revealed due to the restoration of CD4 lymphocytes leading to tissue infiltration and inflammation resembling a T1R [24, 25]. In our study, the seven leprosy patients co-infected with HIV did not present with a higher BI or higher rate of MB disease than non-co-infected individuals. In the 5 out of 7 subjects that were under antiretroviral therapy, 4 patients developed reactions or neuritis. However, we have documented an association with HTLV-1 in three HIV patients under antiretroviral therapy, all presenting reactions or neuritis, suggesting that the interaction between HTLV-1, HIV and antiretroviral therapy is an important predisposing factor for development of reactions or neuritis.

The prevalence of HTLV-1 co-infection in leprosy varies widely, from 37% in Congo [26] to 0.4% in Ethiopia [27] suggesting that the association between these pathogens is stronger in endemic regions for both agents. HTLV-1 infection is associated with the release of inflammatory cytokines and with a decrease in T-cell immunity by Th2 cytokine production [28]. However, several studies of HTLV-1 co-infection in leprosy did not refer to any association with clinical outcomes [20]. A case report from Brazil describes the development of a T2R in a subject presenting with HTLV-1 associated myelopathy/tropical spastic paraparesis (HAM/TSP) [29]. We found that co-infection with HTLV-1 is associated with a high rate of unfavorable leprosy outcomes; all co-infected patients had complications, and predominately acute inflammatory episodes (T1R, T2R and neuritis). The only subject without reactions or neuritis developed a silent neuropathy with a disability grade 1. It is interesting to note that the highest frequency of T1R plus T2R as well as neuritis was found in the HTLV-1 co-infected group, suggesting that the host immune response varies widely over time in the same individual, and also that the peripheral nerves are a main target.

A higher prevalence of HTLV-1 (2.5%) was found in our cohort of outpatients, as compared to the general population in Bahia where the rate is 1.8% [30]. Similarly we have found a higher prevalence of HBV exposure (10.6%), compared to rates that range from 3% (general population) to 8% (health workers) in Bahia [31]. This data suggests that leprosy subjects may be at higher a risk for infection by these viruses. Alternatively, HTLV-1 or HBV exposure may preceded leprosy and predispose to its development by interfering with cellular immune mechanisms. Due to the long period of incubation of leprosy and the absence of an identifiable risk factor for acquiring those viral co-infections in our cohort, it is not possible to determine which came first, leprosy or viral co-infection.

Our data show a general high rate of reactions, neuritis and neuropathy that may be explained by the inclusion of subjects from two reference leprosy centers in Bahia where complicated cases are referred. It is interesting to note that T2Rs were more frequent among non-co-infected (32.5%) compared with co-infected subjects (19.4%). These data raise a speculative question about a possible “protective” role of viral co-infection against T2R.

Although we did not find any major differences regarding BI and expression of MB disease in the groups, we did also document an impressive relapse rate in the co-infected subjects, implying that these patients should be carefully monitored during and after MDT.

Our study has limitations. The sample size was relatively small, which limited the statistical power for subgroup analyses. We also cannot determine whether the viral co-infections were causally related to the adverse outcomes or merely markers of unknown factors that are truly causal.

In summary, our data shows that HBV, HCV, HIV and HTLV-1 co-infections may be associated with many unfavorable outcomes in leprosy subjects, from higher number of inflammatory complications and nerve damage to an increased relapse rate. Therefore, determining the serological status of leprosy patients for HBV, HCV, HIV and HTLV-1 should be considered as an important standard of care and implemented in regions where any of these viruses are endemic.

## Supporting Information

S1 Checklist STROBE checklist. (DOCX)
