# Supplementary material for: Viral Co-infection and Leprosy Outcomes: A Cohort Study
Source: PLoS Negl Trop Dis. 2015 Aug 12;9(8):e0003865. doi: 10.1371/journal.pntd.0003865 (PMC4534371; doi:10.1371/journal.pntd.0003865)
Supplement: S1 Checklist — (DOCX) [file pntd.0003865.s001.docx]

STROBE Statement—Checklist of items that should be included in reports of ***cohort studies***

|  | Item No | Recommendation |
| --- | --- | --- |
| **Title and abstract** | 1 | (*a*) Indicate the study’s design with a commonly used term in the title or the abstract  – Indicated in the title and abstract. |
|  |  | (*b*) Provide in the abstract an informative and balanced summary of what was done and what was found  - Done |
| Introduction | | |
| Background/rationale | 2 | Explain the scientific background and rationale for the investigation being reported  - It is found at pages 3 and 4 |
| Objectives | 3 | State specific objectives, including any prespecified hypotheses  - It is found at pages 3 and 4 |
| Methods | | |
| Study design | 4 | Present key elements of study design early in the paper  - It is found at page 4, in the “Study Design” section |
| Setting | 5 | Describe the setting, locations, and relevant dates, including periods of recruitment, exposure, follow-up, and data collection  - It is found at page 4, in the “Study Design” section |
| Participants | 6 | (*a*) Give the eligibility criteria, and the sources and methods of selection of participants. Describe methods of follow-up  - It is found at pages 4 and 5, in the “Study Design” and “Inclusion Criteria” sections |
|  |  | (*b*) For matched studies, give matching criteria and number of exposed and unexposed  - Not applicable |
| Variables | 7 | Clearly define all outcomes, exposures, predictors, potential confounders, and effect modifiers. Give diagnostic criteria, if applicable  - It is found at page 5, in the “Outcome Definitions” section |
| Data sources/ measurement | 8* | For each variable of interest, give sources of data and details of methods of assessment (measurement). Describe comparability of assessment methods if there is more than one group  - It is found at pages 4, 5 and 6, in the “Study Design”, “Outcome Definitions” and “Serology” sections |
| Bias | 9 | Describe any efforts to address potential sources of bias  - It is found at pages 4 and 5, in the “Study Design” and “Outcome Definitions” sections, and discussed at page 15 |
| Study size | 10 | Explain how the study size was arrived at  - It is found at page 6, in the “Sample size” section |
| Quantitative variables | 11 | Explain how quantitative variables were handled in the analyses. If applicable, describe which groupings were chosen and why  - It is found at page 6, in the “Statistical Analysis” section |
| Statistical methods | 12 | (*a*) Describe all statistical methods, including those used to control for confounding  - It is found at page 6, in the “Statistical Analysis” section |
|  |  | (*b*) Describe any methods used to examine subgroups and interactions  - It is found at page 6, in the “Statistical Analysis” section |
|  |  | (*c*) Explain how missing data were addressed  - Not applicable |
|  |  | (*d*) If applicable, explain how loss to follow-up was addressed  - It is found at page 7, in the “Results” section |
|  |  | (*e*) Describe any sensitivity analyses  - Not applicable |
| Results | | |
| Participants | 13* | (a) Report numbers of individuals at each stage of study—eg numbers potentially eligible, examined for eligibility, confirmed eligible, included in the study, completing follow-up, and analysed  - It is found at page 7, in the “Results” section |
|  |  | (b) Give reasons for non-participation at each stage  - It is found at page 7, in the “Results” section |
|  |  | (c) Consider use of a flow diagram  - Not applicable |
| Descriptive data | 14* | (a) Give characteristics of study participants (eg demographic, clinical, social) and information on exposures and potential confounders  - It is found at page 7, in the “Results” section and table 1 |
|  |  | (b) Indicate number of participants with missing data for each variable of interest  - Not applicable |
|  |  | (c) Summarise follow-up time (eg, average and total amount)  - It is found at page 8, in the “Results” section and table 1 |
| Outcome data | 15* | Report numbers of outcome events or summary measures over time  - It is found at page 8 and 9, in the “Results” section and table 2 |
| Main results | 16 | (*a*) Give unadjusted estimates and, if applicable, confounder-adjusted estimates and their precision (eg, 95% confidence interval). Make clear which confounders were adjusted for and why they were included  - It is found at pages 7 to 9 in tables 1 and 2 |
|  |  | (*b*) Report category boundaries when continuous variables were categorized  - It is found at pages 7 to 9 in tables 1 and 2 |
|  |  | (*c*) If relevant, consider translating estimates of relative risk into absolute risk for a meaningful time period  - Not applicable |
| Other analyses | 17 | Report other analyses done—eg analyses of subgroups and interactions, and sensitivity analyses  - Not applicable |
| Discussion | | |
| Key results | 18 | Summarise key results with reference to study objectives  - It is found at pages 8 to 10 in the “Results” section |
| Limitations | 19 | Discuss limitations of the study, taking into account sources of potential bias or imprecision. Discuss both direction and magnitude of any potential bias  - It is found at page 15, in the “Discussion” section |
| Interpretation | 20 | Give a cautious overall interpretation of results considering objectives, limitations, multiplicity of analyses, results from similar studies, and other relevant evidence  - It is found at pages 14 and 15, in the “Discussion” section |
| Generalisability | 21 | Discuss the generalisability (external validity) of the study results  - It is found at page 15, in the “Discussion” section |
| Other information | | |
| Funding | 22 | Give the source of funding and the role of the funders for the present study and, if applicable, for the original study on which the present article is based  - It is included in the financial disclosure section of the online submission system. |

*Give information separately for exposed and unexposed groups.

**Note:** An Explanation and Elaboration article discusses each checklist item and gives methodological background and published examples of transparent reporting. The STROBE checklist is best used in conjunction with this article (freely available on the Web sites of PLoS Medicine at http://www.plosmedicine.org/, Annals of Internal Medicine at http://www.annals.org/, and Epidemiology at http://www.epidem.com/). Information on the STROBE Initiative is available at http://www.strobe-statement.org.
